# Kinetic model of radiochemical oxygen depletion (ROD) in FLASH radiotherapy

**DOI:** 10.1002/mp.70383

**Published:** 2026-03-25

**Authors:** Joao Seco, Hugo Freitas

**Affiliations:** ^1^ Division of Biomedical Physics in Radiation Oncology DKFZ, German Cancer Research Centre Heidelberg Germany; ^2^ Department of Physics and Astronomy University of Heidelberg Heidelberg Germany

**Keywords:** FLASH, oxygen consumption, radiochemical oxygen depletion, ROD

## Abstract

**Background:**

The role of oxygen in “Ultra‐High Dose Rate” (UHDR) radiotherapy is currently subject to active debate, due to its importance in the FLASH effect. Radiochemical oxygen depletion (ROD) is used to characterize the removal of oxygen by its interaction with the free radicals produced by the radiation. Currently, there is a need to understand why ROD depends on the radiation dose rate and the initial oxygen pressure.

**Purpose:**

Development of a kinetic model of ROD that explains its dependence on (i) radiation dose rate and (ii) initial oxygen pressure, $O_{2}$.

**Methods:**

The current work uses a variety of published ROD studies performed in vitro and in vivo in mice to evaluate the kinetic model prediction of ROD. The in vitro studies include evaluation of ROD in water, bovine serum albumin (BSA), and CELL medium consisting of HEPES (10mM), glycerol (1M), glucose (5mM), and glutathione (5mM). Published in vivo studies were performed in C57BL/6 mice (male and female) and NU(Ico)‐Foxn1nu mice (female Swiss nude) using proton FLASH and electron, respectively. Oxygen pressure measurements were performed with a variety of different probes such as (i) TROXSP5 sensors, (ii) Oxyphor PtG4, and (iii) Oxylite (NX‐BF/OT/E). Two definitions of ROD were used in the current work to represent separately the ROD dependence in “time” ($\mathrm{RO}D_{\mathrm{Time}}$) and “dose” ($\mathrm{RO}D_{\mathrm{Dose}}$).

**Results:**

The kinetic model $\mathrm{RO}D_{\mathrm{Dose}}$ prediction agreed well with published measurements, yielding reduced $\chi^{2}$ values near the unity for water, BSA, and CELL medium, and comparably strong agreement for the animal‐study datasets, within the reported or estimated uncertainties used in this work. The solvated electron *G*‐value, $G_{e_{\mathrm{aq}}^{-}}$, was shown to be dose rate, LET dependent and medium specific. For a medium with radical scavenging capacity (such as BSA and CELL), a higher value of $G_{e_{\mathrm{aq}}^{-}}$ was observed compared to water, which had a much lower radical scavenging capacity. The kinetic model $\mathrm{RO}D_{\mathrm{Dose}}$ dose rate predictions also achieved very good agreement, with the published in vitro in water and BSA medium. The $\mathrm{RO}D_{\mathrm{Dose}}$ kinetic model's dose‐rate predictions for in vivo mice studies also showed excellent agreement once the raw oxygen consumption data were corrected for oxygen diffusion during radiation delivery.

**Conclusions:**

A systematic review of all published ROD studies was performed and used as the basis for testing the novel kinetic model for ROD. The kinetic model prediction of ROD showed that the radiolysis products, OH•, $e_{\mathrm{aq}}^{•-}$, $O_{2}^{•-}$, ${\mathrm{HO}}_{2}^{•-}$, play an important role in ROD and provide an explanation why ROD depends on (1) dose rate and (2) initial oxygen pressure.

## 1 INTRODUCTION

The application of ultra‐high dose rates (UHDR, >40Gy/s) has attracted considerable attention in recent years, when compared to conv‐RT (given at 2Gy/min) due to more than 50% reduction in toxicity in healthy lung tissue of mice.^1^ In the same experiment, the radiation induced tumor growth delay remained comparable between UHDR and conv‐RT. The observed differential response between increased normal tissue sparing and equal tumor control was coined the “FLASH effect”. In the current document, we call “FLASH‐RT”, the delivery of radiation at UHDR.

Several possible mechanisms have been proposed to explain the FLASH effect such as oxygen depletion, radical recombination, immune response, etc.^2^ Oxygen is one of the most effective dose‐modifying agents, sensitizing cells to radiation, whereas during FLASH‐RT oxygen damage is significantly reduced. Oxygen causes an “amplification” of radiation‐induced DNA damage, which is commonly described by the oxygen enhancement ratio (OER). The OER varies between 1 and approximately 3, respectively, when going from hypoxic (no oxygen) conditions to normoxic conditions.

The first mammalian studies of UHDR irradiation using electron beams were performed by Town^3^ and Berry et al.^4^ who demonstrated that cells behaved as if hypoxic, even though oxygen was present in the medium during UHDR irradiation. One of the first indirect in vitro assessments of the oxygen effect during UHDR irradiation was performed by Weiss et al.,^5^ where the oxygen conditions for the cells were set before irradiation. The first direct online in vitro measurements of oxygen during UHDR irradiation were performed by Jansen et al.^6^ using TROXSP5 sensors with an infrared‐based readout system and placed within an air‐tight water filled plastic phantom. The obtained correlations of dose rate and oxygen depletion provide some evidence against the oxygen depletion hypothesis as sole explanation of the Flash effect. The first direct online in vivo was performed by Cao et al.^7^ using the injectable phosphorescence probe “Oxyphor 2P” to measure tissue oxygenation in dependence on electron dose rate. This group observed a low level of oxygen consumption by UHDR probably not sufficient for normal tissue protection above hypoxia level.

In the current work, we review published oxygen depletion studies performed in vitro and in vivo for water, BSA, CELL, and in mice^6^, ^7^, ^8^, ^9^, ^10^, ^11^, ^12^ and compare them to the predictions of a novel kinetic model for radiochemical oxygen depletion (ROD). The ROD behavior during irradiation is derived from the chemical reaction equations of oxygen and the reactive species produced by water radiolysis, such as OH•, $e_{\mathrm{aq}}^{•-}$, H•, $H_{2}O_{2}$, $H_{2}$, $O_{2}^{•-}$, and ${\mathrm{HO}}_{2}^{•-}$.

### 1.1 Radiolysis reactions

Ionizing radiation causes radiolysis of water molecules, resulting in the production of a variety of reactive species (OH•, $e_{\mathrm{aq}}^{•-}$, H•, $H_{2}O_{2}$, and $H_{2}$), with additional reactive species produced in the presence of oxygen ($O_{2}^{•-}$ and ${\mathrm{HO}}_{2}^{•-}$). In Table 1, we provide the main reactions between the various reactive species. Table 1 presents the reactions along with the rates of production of primary radiolysis species. The rates are expressed as the product of G‐values and the radiation dose rate. The table also includes the key reactions among the radiolysis products, relevant hydrolysis reactions, and associated acid–base equilibria, at temperatures of 25 and $37^{\circ }C$. The rate constants for in vitro and in vivo conditions are taken at 25 and $37^{\circ }C$, respectively. This reaction set has been published previously (Wren and Ball^13^), where additional temperature dependence of the rates constants can be found in Buxton et al.,^14^ Elliot and Buxton,^15^ Elliot,^16^ and Sunaryo et al.^17^

**TABLE 1:** Radiolysis reactions between the various reactive species at 25 and $37^{\circ }C$.

Reaction no.	*G*‐values (Units of molecules/100 eV) Formation reaction	*G*‐value	
R1a	$H_{2}O\to e_{\mathrm{eq}}^{•-}$	2.6	
R1b	$H_{2}O\to H^{+}$	2.6	
R1c	$H_{2}O\to H^{•}$	0.6	
R1d	$H_{2}O\to OH^{•}$	2.7	
R1e	$H_{2}O\to H_{2}$	0.45	
R1f	$H_{2}O\to H_{2}O_{2}$	0.7	

	Chemical reaction	Rate constant (25C)	Rate constant (37C)
	$e_{•-}^{\mathrm{eq}}$		
R2	$e_{\mathrm{eq}}^{•-}+e_{\mathrm{eq}}^{•-}+2H_{2}O\to H_{2}+2OH^{-}$	$5.5\times 10^{9}$	$7.55\times 10^{9}$
R3	$e_{\mathrm{eq}}^{•-}+H^{•}+H_{2}O\to H_{2}+OH^{-}$	$2.5\times 10^{10}$	$3.22\times 10^{10}$
R4	$e_{\mathrm{eq}}^{•-}+OH^{•}\to OH^{-}$	$3.0\times 10^{10}$	$3.39\times 10^{10}$
R5	$e_{\mathrm{eq}}^{•-}+O_{2}\to O_{2}^{•-}$	$2.22\times 10^{10}$	$2.77\times 10^{10}$
R6	$e_{\mathrm{eq}}^{•-}+H_{2}O_{2}\to OH^{-}+OH^{•}$	$1.6\times 10^{10}$	$2.03\times 10^{10}$
R7	$e_{\mathrm{eq}}^{•-}+{\mathrm{HO}}_{2}^{•}\to {\mathrm{HO}}_{2}^{-}$	$1.3\times 10^{10}$	$1.61\times 10^{10}$
	$H^{•}$		
R8	$H^{•}+OH^{•}\to H_{2}O$	$7\times 10^{9}$	$7.9\times 10^{9}$
R9	$H^{•}+H^{•}\to H_{2}$	$7.75\times 10^{9}$	$9.8\times 10^{9}$
R10	$H^{•}+O_{2}\to {\mathrm{HO}}_{2}^{•}$	$2.1\times 10^{10}$	$2.48\times 10^{10}$
R11	$H^{•}+{\mathrm{HO}}_{2}^{•}\to H_{2}O_{2}$	$1.0\times 10^{10}$	$1.18\times 10^{10}$
R12	$H^{•}+H_{2}O_{2}\to OH^{•}+H_{2}O$	$9.0\times 10^{7}$	$1.15\times 10^{8}$
R13	$H^{•}+O_{2}^{•-}\to {\mathrm{HO}}_{2}^{-}$	$2.0\times 10^{10}$	$2.36\times 10^{10}$
	$OH^{•}$		
R14	$OH^{•}+OH^{•}\to H_{2}O_{2}$	$5.5\times 10^{9}$	$6.2\times 10^{9}$
R15	$OH^{•}+H_{2}O_{2}\to {\mathrm{HO}}_{2}^{•}+H_{2}O$	$2.7\times 10^{7}$	$3.45\times 10^{7}$
R16	$OH^{•}+H_{2}\to H^{•}+H_{2}O$	$4.2\times 10^{7}$	$5.58\times 10^{7}$
R17	$OH^{•}+O_{2}^{•-}\to OH^{-}+O_{2}$	$8\times 10^{9}$	$9.48\times 10^{9}$
R18	$OH^{•}+{\mathrm{HO}}_{2}^{•}\to H_{2}O+O_{2}$	$6\times 10^{9}$	$6.55\times 10^{9}$
	$HO_{•}^{2}$		
R19	${\mathrm{HO}}_{2}^{•}+O_{2}^{•-}\to {\mathrm{HO}}_{2}^{-}+O_{2}$	$8.9\times 10^{7}$	$1.02\times 10^{8}$
R20	${\mathrm{HO}}_{2}^{•}+{\mathrm{HO}}_{2}^{•}\to H_{2}O_{2}+O_{2}$	$2.0\times 10^{6}$	$2.69\times 10^{6}$

	Related equilibrium reaction	Rate constant	
R21	${\mathrm{HO}}_{2}^{•}⇌O_{2}^{•-}+H^{+}$	$1.5\times 10^{-5}$ or $pK_{a}$ = 4.82	

The rates of production of primary radiolysis species (*G*‐values, given in units of molecules/100eV) can be converted to a concentration per dose rate, via the constant $C_{R}$ and defined in Joseph et al.^18^ by the following expression $C_{R}=1.04\times 10^{-7}\;\mathrm{mol}/L\times \frac{1}{G-\mathrm{value}}\times \frac{1}{Gy/s}$ and dose rate $\dot{D}$. For example, for $H_{2}$ ($G_{H_{2}}=0.45$) we have ${\left[H_{2}\right]}_{G-\mathrm{value}}=0.45\times 1.04\times 10^{-7}\times \dot{D}\times \mathrm{mol}/L=0.468\times 10^{-7}\dot{D}M$, where M=mol/L.

The kinetic model that quantifies oxygen removal via the reactions with radiolysis products, can be built using the reactions listed in Table 1 (no diffusion is included):

(1) $$\begin{array}{cc} \frac{\partial \left[O_{2}\right]}{\partial t} & =-k_{R5}.\left[O_{2}\right].\left[e_{\mathrm{aq}}^{•-}\right]+k_{R17}.\left[\mathrm{OH}•\right].\left[O_{2}^{•-}\right]+k_{R18}\\ & \;.\left[\mathrm{OH}•\right].\left[{\mathrm{HO}}_{2}^{•-}\right]+k_{R19}.\left[{\mathrm{HO}}_{2}^{•-}\right].\left[O_{2}^{•-}\right]+k_{R20}\\ & \;.\left[{\mathrm{HO}}_{2}^{•-}\right].\left[{\mathrm{HO}}_{2}^{•-}\right] \end{array}$$

where $O_{2}$ represents the oxygen pressure in the medium, that is time dependent, and $\left[O_{2}\right]$ represents the concentration of oxygen that is dissolved in the medium. Since the initial concentration of oxygen is usually much higher than the primary radiolysis species, the dominant term in Equation (1) is reaction R5, leading to the following approximate solution:

(2) $$\frac{\partial \left[O_{2}\right]}{\partial t}≅-k_{R5}.\left[O_{2}\right].\left[e_{\mathrm{aq}}^{•-}\right]$$

Oxygen consumption is thus primarily driven by interaction of oxygen with the solvated electron, $e_{\mathrm{aq}}^{•-}$. The dose rate dependency of solvated electron, $e_{\mathrm{aq}}^{•-}$, in water radiolysis was shown experimentally by Zhang et al.,^19^ where UHDR radiation produces less hydrogen peroxide ($H_{2}O_{2}$) and less oxygen depletion in pure water. The reduced oxygen depletion observed during UHDR radiation delivery compared to standard dose‐rate delivery may help explain the protective nature of the FLASH effect.

### 1.2 Terminology: Radiochemical oxygen depletion—ROD

The term ROD is commonly used to characterize the removal of oxygen by its interaction with the free radicals present in the medium. In the current manuscript we will define a ROD per unit time, $\mathrm{RO}D_{\mathrm{Time}}$ and ROD per unit dose, $\mathrm{RO}D_{\mathrm{Dose}}$, both given by the following expressions:

(3) $$\mathrm{RO}D_{\mathrm{Time}}=-\frac{\partial \left[O_{2}\right]}{\partial t}$$

(4) $$\mathrm{RO}D_{\mathrm{Dose}}=-\frac{\partial \left[O_{2}\right]}{\partial D}$$

where $\mathrm{RO}D_{\mathrm{Time}}=\dot{D}\times \mathrm{RO}D_{\mathrm{Dose}}$. We use these two definitions to represent separately the ROD dependence in “time” and “dose”.

### 1.3 Steady state solutions for the reactive chemical species

The time‐dependent concentration of the various reactive species in an irradiated water sample, undergoing irradiation, can be calculated using the set of reactions listed in Table 1. The concentrations of the radiolysis products, in general, quickly reach steady state, since, for a given species, the main production path is the primary radiolysis process whose rate is assumed constant during radiation delivery, while the removal process is typically via fast radical–molecule or ion–molecule reactions. Under these conditions, steady‐state kinetic analysis can be applied to a smaller sub‐set consisting of the main production and removal reactions, if they can be identified, to determine approximately the concentrations of the radiolysis products.

The exact solution to the time‐dependent chemical reactions is given in Joseph et al.,^18^ where they are compared to the stead‐state solutions. We use the steady state solutions from Joseph et al.^18^ to develop a kinetic model of the effect of UHDR on oxygen depletion.

The steady state solution for solvated electron is given by^20^:

(5) $$\begin{array}{cc} & {\left[e_{\mathrm{aq}}^{•-}\right]}_{\mathrm{SS}}\\ & ≅\frac{C_{R}\;G_{e_{\mathrm{aq}}^{•-}}\dot{D}}{k_{R4}{\left[\mathrm{OH}•\right]}_{\mathrm{SS}}+k_{R5}\left[O_{2}\right]+k_{R6}{\left[H_{2}O_{2}\right]}_{\mathrm{SS}}+k_{R7}{\left[{\mathrm{HO}}_{2}^{•}\right]}_{\mathrm{SS}}} \end{array}$$

where $\dot{D}$ is the radiation dose rate, $G_{e_{\mathrm{aq}}^{•-}}$ is the *G*‐value for the solvated electron, and SS stands for “*steady‐state”*. Equation (5) encompasses all reactions involved in the radiolysis of pure water, including the formation rates of primary radiolysis species (expressed as the product of *G*‐values and radiation dose rate), as well as all subsequent reactions among the radiolysis products, hydrolysis processes, and the associated acid–base equilibria described in Table 1. The steady state solution for the solvated electron depends on the concentrations of OH•, $O_{2},$ $H_{2}O_{2}$, and ${\mathrm{HO}}_{2}^{•}$. The solvated *G*‐value, $G_{e_{\mathrm{aq}}^{•-}}$, represents the production of solvated electrons by the radiation during water radiolysis, and is non‐zero only during radiation delivery.^20^

The full‐model steady state predictions from Joseph et al.,^18^ for the reactive species OH•, $H_{2}O_{2}$, ${\mathrm{HO}}_{2}^{•}$, are the following:

(6) $${\left[\mathrm{OH}•\right]}_{\mathrm{SS}}≅\frac{C_{R}G_{\mathrm{OH}•}\dot{D}}{k_{R17}}\sqrt{\frac{k_{R19}\left[H^{+}\right]}{k_{R21}^{\mathrm{eq}}C_{R}\;G_{e_{\mathrm{aq}}^{•-}}\dot{D}}}$$

(7) $${\left[H_{2}O_{2}\right]}_{\mathrm{SS}}≅\frac{C_{R}G_{H_{2}O_{2}}\dot{D}+k_{R19}{\left[O_{2}^{•-}\right]}_{\mathrm{SS}}{\left[{\mathrm{HO}}_{2}^{•-}\right]}_{\mathrm{SS}}}{k_{R6}{\left[e_{\mathrm{aq}}^{•-}\right]}_{\mathrm{SS}}+k_{R15}{\left[\mathrm{OH}•\right]}_{\mathrm{SS}}}$$

(8) $${\left[{\mathrm{HO}}_{2}^{•}\right]}_{\mathrm{SS}}≅\sqrt{\frac{k_{R5}\left[H^{+}\right]}{k_{R19}k_{R21}^{\mathrm{eq}}}{\left[e_{\mathrm{aq}}^{•-}\right]}_{\mathrm{SS}}{\left[O_{2}\right]}_{\mathrm{SS}}}$$

Equation (6) shows that ${\left[\mathrm{OH}•\right]}_{\mathrm{SS}}$ has an approximate $\sqrt{\dot{D}}$ dose rate dependence and previously observed for water UHDR studies by Jansen et al.^6^ This behavior is primarily driven by the equilibrium reaction $R_{21}$ which establishes a very close coupling between ${\left[{\mathrm{HO}}_{2}^{•}\right]}_{\mathrm{SS}}$ and ${\left[O_{2}^{•-}\right]}_{\mathrm{SS}}$, and the reactions (1) $R_{17}$, which leads to the removal of the hydroxyl radical (OH•) by the superoxide ($O_{2}^{•-}$), in the presence of oxygen and (2) $R_{19}$ ***_,_*** which leads to the removal of both ${\mathrm{HO}}_{2}^{•}$ and $O_{2}^{•-}$. In addition, in Equation (5) we note that all reactive species (OH•, $H_{2}O_{2}$, ${\mathrm{HO}}_{2}^{•}$) have an effect on the total solvated electron concentration. However, $e_{\mathrm{aq}}^{•-}$ has the highest reaction rate with OH•, $3.0\times 10^{10}$($3.39\times 10^{10}$), compared to $H_{2}O_{2}$, ${\mathrm{HO}}_{2}^{•}$, which are $1.6\times 10^{10}$ ($2.03\times 10^{10}$) and $1.3\times 10^{10}$ ($1.61\times 10^{10}$) respectively at the temperatures $25^{\circ }C$ ($37^{\circ }C$).

### 1.4 Kinetic model definition of $\mathrm{RO}D_{\mathrm{Time}}$ and $\mathrm{RO}D_{\mathrm{Dose}}$

The novel kinetic model $\mathrm{RO}D_{\mathrm{Time}}$ proposed in the current manuscript can now be defined by combing Equations (2) and (5), which yields:

(9) $$\begin{array}{ccc} & & \mathrm{RO}D_{\mathrm{Time}}=-\frac{\partial \left[O_{2}\right]}{\partial t}≅k_{R5}.\left[O_{2}\right]\\ & & \cdot \frac{C_{R}G_{e_{\mathrm{aq}}^{•-}}\dot{D}}{k_{R4}{\left[\mathrm{OH}•\right]}_{\mathrm{SS}}+k_{R5}\left[O_{2}\right]+k_{R6}{\left[H_{2}O_{2}\right]}_{\mathrm{SS}}+k_{R7}{\left[{\mathrm{HO}}_{2}^{•}\right]}_{\mathrm{SS}}} \end{array}$$

Since $k_{R4}>k_{R6}>k_{R7}$, ${\left[\mathrm{OH}•\right]}_{\mathrm{SS}}\gg {\left[H_{2}O_{2}\right]}_{\mathrm{SS}}$ and ${\left[\mathrm{OH}•\right]}_{\mathrm{SS}}\gg {\left[{\mathrm{HO}}_{2}^{•}\right]}_{\mathrm{SS}}$ we approximate the previous equation to the following (using Equation 6):

(10) $$\begin{array}{cc} \mathrm{RO}D_{\mathrm{Time}} & =k_{R5}.\left[O_{2}\right].\frac{C_{R}G_{e_{\mathrm{aq}}^{•-}}\dot{D}}{k_{R5}\left[O_{2}\right]+k_{R4}{\left[\mathrm{OH}•\right]}_{\mathrm{SS}}}\\ & =\frac{C_{R}G_{e_{\mathrm{aq}}^{•-}}\left[O_{2}\right]\dot{D}}{\left[O_{2}\right]+K\sqrt{\dot{D}}} \end{array}$$

where $K=\frac{k_{R4}C_{R}G_{\mathrm{OH}•}}{k_{R5}k_{R17}}\;\sqrt{\frac{k_{R19}\left[H^{+}\right]}{k_{R21}^{\mathrm{eq}}C_{R}G_{e_{\mathrm{aq}}^{•-}}}}$. The $\mathrm{RO}D_{\mathrm{Time}}$ expression can now be converted into $\mathrm{RO}D_{\mathrm{Dose}}$, yielding the following expression:

(11) $$\mathrm{RO}D_{\mathrm{Dose}}=-\frac{\partial \left[O_{2}\right]}{\partial D}≅\frac{C_{R}G_{e_{\mathrm{aq}}^{•-}}\left[O_{2}\right]}{\left[O_{2}\right]+K\sqrt{\dot{D}}}$$

The $\mathrm{RO}D_{\mathrm{Dose}}$ has the same functional expression as the Michaelis–Menten formula, with Michaelis–Menten parameters $V_{\max}=C_{R}\;G_{e_{\mathrm{aq}}^{•-}};$ $K_{M}=K\sqrt{\dot{D}}$ and the oxygen function given by $f\left(O_{2}\right)=\frac{\left[O_{2}\right]}{\left[O_{2}\right]+K\sqrt{\dot{D}}}$, which varies between 0 and 1, depending on the oxygen level and the dose‐rate. The Michaelis–Menten parameters ($V_{\max}$ and $K_{m}$) will be used throughout the manuscript for fitting experimental data.

## 2 MATERIALS AND METHODS

### 2.1 ROD studies performed in vitro in water, BSA, and CELL medium

#### 2.1.1 Water (H_2_O) measurements

Published oxygen depletion studies in $H_{2}O$ include experimental measurements from Jansen et al.^6^ and Jansen et al.,^8^ and simulation studies from Boscolo (2021)^21^ (see Table 2). The experimental studies (Jansen et al.^6^, ^8^) included measurements of oxygen depletion in water, with sealed water phantoms (size: 2.5 mm diameter and 5mm length), which were homogeneously irradiated. The oxygen depletion studies were performed using photons, protons, and electrons (see Table 2 for energies used). The oxygen level of the water inside the phantom was modified by inducing a gas‐exchange in double de‐ionized $H_{2}O$ in a hypoxic chamber (Baker Ruskinn) with fixed $O_{2}$ level. The $O_{2}$ measurements were performed using TROXSP5 sensors (PyroScience). The simulation studies (Boscolo (2021)^21^) involved the use of the Monte Carlo code TRAX‐CHEM to simulate 1MeV electrons in oxygenated water with a single pulse delivery of the dose.

**TABLE 2:** Published radiochemical oxygen depletion (ROD) results.

Study	Medium	Oxygen probe	Particle type
Jansen (2021)	$H_{2}O$	TROXSP5 sensors	225kV photons, 224MeV protons
Boscolo (2021)	$H_{2}O$	TRAX‐CHEM sim.	1MeV electrons
Jansen (2022)	$H_{2}O$	TROXSP5 sensors	30MeV electrons
Van Slyke (2022)	CELL	Oxyphor PtG4	230MeV protons
Sunnerberg (2023)	$H_{2}O+$ BSA (4%w/w)	Oxyphor PtG4	10MeV electrons
Karle (2025)	$H_{2}O+$ BSA (5%w/w)	Oxylite (NX‐BF/OT/E)	9MeV Electrons, 146.56MeV protons, 145.74MeV helium ions, 275.98MeV/u carbon and 325.98MeV/u oxygen ions
Van Slyke (2022)	C57BL/6 Mouse (male and female)	Oxyphor PtG4	230MeV protons
Grilj (2024)	NU(Ico)‐Foxn1nu Mouse (female swiss Nude)	Oxyphor PtG4	6MeV electrons

#### 2.1.2 Bovine serum albumin (BSA) and CELL measurements

Published oxygen depletion studies in BSA and CELL include experimental studies from Van Slyke et al.^10^ and Sunnerberg et al.^11^ (see Table 2). The BSA system consists of water with BSA, at a 4% BSA weight per weight of solution (4%w/w). Although the CELL medium consists of HEPES (10mM), glycerol (1M), glucose (5mM), and glutathione (5mM), following the receipt from Ayene et al.^20^ These studies were performed using either electron or proton beams (Table 2). Oxygen partial pressure ($pO_{2}$) measurements were performed with a phosphorescent probe Oxyphor PtG4.

### 2.2 ROD studies performed in vivo in mice

Published oxygen depletion studies in vivo include Van Slyke et al.^10^ and Grilj et al.^12^ The Van Slyke et al.^10^ studies were done with eight to 10‐week‐old female C57BL/6 mice. At 24 h prior to irradiation and oxygen measurements, the right thigh muscle or thigh tumor of each control or tumor‐bearing mouse was injected with 20μL of 100μM phosphorescent probe (Oxyphor PtG4). Mice were anesthetized for all oxygenation measurements and irradiations using 1.5% isoflurane in atmospheric gas supplied through a nose cone. Tissue oxygen levels were monitored for several minutes before, during and after irradiation to gather a baseline for tissue oxygenation under anesthesia. The Grilj et al.^12^ studies were done with female Swiss Nude mice.

### 2.3 Oxygen diffusion modeling for oxygen consumption studies performed in mice

**FIGURE 1:**
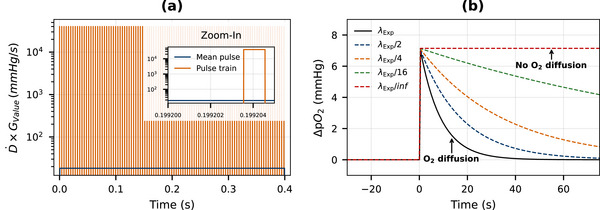
Pulse train (a) used for $\dot{D}=101\;\mathrm{Gy}/s$ delivery and oxygen consumption corrected for diffusion (b).

In the study by Grilj et al.,^12^ oxygen consumption (defined as $\Delta pO_{2}$) was measured in mice for the tumor and normal tissues (skin, muscle, and brain) during radiation delivery. Unlike in vitro studies, in vivo measurements of oxygen consumption are complicated by oxygen diffusion within the tissue during radiation delivery. As a result, the measured signal reflects not only the intrinsic oxygen consumption (without diffusion) but also the contribution from oxygen diffusion within the tissue. The diffusion component represents mostly the movement of oxygen from regions outside the irradiated volume into the irradiated volume. In this study, oxygen diffusion ($O_{2}$_(diffusion)_) was modeled as a Gaussian diffusion process, in which the temporal change within the irradiated tissue follows an exponential recovery pattern, $e^{-\lambda_{\mathrm{Exp}}*t}$, and “Exp” is the measured experimental temporal‐decay at the highest dose rate of 101Gy/s. Consequently, the measured oxygen consumption is related to the intrinsic oxygen consumption (without diffusion) through a convolution with the exponential function that characterizes the diffusion process (see Equation 12).

(12) $$\begin{array}{cc} & \left|\Delta pO_{2\;\left(\mathrm{measured}\right)}-\Delta pO_{2\;\left(\mathrm{baseline},\mathrm{before}\;\mathrm{radiation}\right)}\right|\\ & =\Delta pO_{2\;\left(\mathrm{no}\;\mathrm{diffusion}\right)}⊗O_{2\;\left(\mathrm{diffusion}\right)} \end{array}$$

where the brackets represent the modulus of the difference of the measured $\Delta pO_{2}$ relative to the baseline before radiation. The pulse structure of the radiation delivery was also modeled to accurately represent both oxygen consumption during irradiation and oxygen diffusion during periods when the radiation was on and off. Figure 1 illustrates the pulse train used for delivery at the highest dose rate of 101Gy/s, where the amount of oxygen consumed is directly proportional to the dose rate and the *G*‐value of the solvated electron.

In addition, Figure 1 also shows the modeled oxygen consumption progressively corrected for oxygen diffusion by reducing the diffusion parameter, “$\lambda_{\mathrm{Exp}}$” to zero. The diffusion parameter, “$\lambda_{\mathrm{Exp}}$”, obtained at the highest dose rate of 101Gy/s was then used to correct the in vivo oxygen consumption data for the dose rates of 2, 4, 8, 20, and 40Gy/s. Since both oxygen consumption and oxygen diffusion is tissue specific, a diffusion parameter must be obtained for each tissue at the highest dose rate.

### 2.4 Linear energy transfer (LET) impact on the *G*‐value of the solvated electron ($G_{e_{\mathrm{aq}}^{•-}}$)

LET and oxygen consumption are critical parameters for understanding radiation response. LET defines the density of energy deposition along the radiation track, influencing both the yield of solvated electrons (quantified by $G_{e_{\mathrm{aq}}^{•-}}$) and the extent of oxygen consumption. Oxygen consumption data as a function of initial oxygen pressure from Karle (2025)^22^ were used to evaluate the effect of LET on ROD. Sealed samples containing 5% BSA were irradiated with 15Gy using electrons, protons, helium, carbon, and oxygen ions, corresponding to LET*_d_* values of 1, 5.4, 14.4, 65, and 100.3keV/μm, respectively. Oxygen consumption was evaluated at both conventional and UHDR of 0.3−0.4Gy/s and 100Gy/s. Oxygen concentration was measured before and after irradiation using the Oxylite system, enabling calculation of the oxygen consumption rate.

### 2.5 Data analysis and curve fitting

Data from the studies presented in Table 2 were fitted with the kinetic model described by Equation (10) for the $\mathrm{RO}D_{\mathrm{Time}}$ or Equation (11) for $\mathrm{RO}D_{\mathrm{Dose}}$. Data fitting was performed using weighted least squares under the assumption of Gaussian uncertainties, by minimizing the chi‐square statistic, $\chi^{2}=\sum_{i=1}^{n}{\left(\frac{y_{i}-\mathrm{RO}D_{\mathrm{model}}\left(x_{i}\right)}{\sigma_{i}}\right)}^{2}$, where $y_{i}$ are the published experimental data, $\mathrm{RO}D_{\mathrm{model}}$ is the model prediction, and $\sigma_{i}$ are the uncertainties. For literature datasets that do not report measurement uncertainties, a conservative 10% relative systematic uncertainty was assigned to all points to enable goodness‐of‐fit evaluation. For datasets that report statistical error bars but omit systematic components, an additional 5% relative uncertainty was added in quadrature to the reported errors. The resulting goodness‐of‐fit was assessed using the reduced chi‐square $\chi_{r}^{2}=\chi^{2}/\nu$, with ν=n−2 degrees of freedom.

## 3 RESULTS

### 3.1 Kinetic model $\mathrm{RO}D_{\mathrm{Dose}}$ as a function of initial oxygen

**FIGURE 2:**
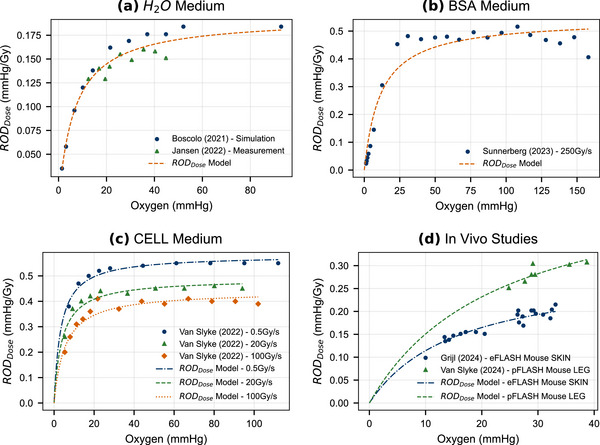
Kinetic model estimation of $\mathrm{RO}D_{\mathrm{Dose}}$ for (a) H_2_O medium, (b) BSA medium, (c) CELL medium, and (d) mouse FLASH irradiation studies with electrons (eFLASH) and protons (pFLASH). BSA, bovine serum albumin; ROD, radiochemical oxygen depletion.

The kinetic model estimate of $\mathrm{RO}D_{\mathrm{Dose}}$ is fitted to the published data (Table 2), for $H_{2}O$, BSA, CELL medium, and mouse measurements. The comparison between measurements and $\mathrm{RO}D_{\mathrm{Dose}}$ prediction is shown in Figure 2 and Table 3, where the optimal $V_{\max}$ and $K_{m}$ are given in the table for each published data set. For the $H_{2}O$ medium, the Boscolo (2021)^21^ and Jansen (2022)^8^ data were combined to allow fitting to an extended oxygen range.

**TABLE 3:** $\mathrm{RO}D_{\mathrm{Dose}}$ fit to published data in Table 2.

Study	$V_{\max}\;\left(\mathrm{mmHg}/\mathrm{Gy}\right)$ $\mathrm{RO}D_{\mathrm{Dose}}$ model	$G_{e_{\mathrm{aq}}^{•-}}$ $\mathrm{RO}D_{\mathrm{Dose}}$ model	$K_{m}\;\left(\mathrm{mmHg}\right)$ $\mathrm{RO}D_{\mathrm{Dose}}$ model	$\chi_{\mathrm{reduced}}^{2}$
Boscolo (2021), Jansen (2022)	0.19	2.2	6.8	0.37
Van Slyke (2022) 0.5Gy/s	0.58	6.8	3.4	0.08
Van Slyke (2022) 20Gy/s	0.49	5.7	3.9	0.29
Van Slyke (2022) 100Gy/s	0.44	5.0	5.5	0.40
Sunnerberg (2023)	0.54	6.3	11.5	1.44
Van Slyke (2022) Mouse	0.50	5.8	23.3	0.18
Grilj (2024) Mouse	0.30	3.5	16.0	0.31

In the $H_{2}O$, BSA, and CELL medium the kinetic $\mathrm{RO}D_{\mathrm{Dose}}$ showed very good agreement with the measurements, with uncertainty‐weighted reduced $\chi^{2}$ indicating good fits. The animal studies were also consistent with the model.

### 3.2 Characterizing dose rate dependence of $G_{e_{\mathrm{aq}}^{•-}}$

The Van Slyke et al.^10^ study observed that for initial oxygen concentrations between 20mmHg and 100mmHg, the oxygen consumption remained constant. However, in Table 3, the Van Slyke $G_{e_{\mathrm{aq}}^{•-}}$ data for dose rates of 0.5, 20, and 100Gy/s show a decreasing trend in $G_{e_{\mathrm{aq}}^{•-}}$ with increasing dose rate. These values were obtained by fitting the Van Slyke data using $\mathrm{RO}D_{\mathrm{Dose}}$. The dose rate dependency of the solvated electron is expected, since at higher dose rates, the solvated electron has an increased probability of interacting with an increasing number of surrounding radicals, yielding lower final $G_{e_{\mathrm{aq}}^{•-}}$. The solvated electron dose rate dependency is approximately given by the power law $G_{e_{\mathrm{aq}}^{•-}}\;\left(\dot{D}\right)=G_{e_{\mathrm{aq}}^{•-}}\;\left(0\right).{\dot{D}}^{-0.0579}$. The dose rate dependency for $G_{e_{\mathrm{aq}}^{•-}}$ value is accounted for in the remaining part of the study when performing ROD modeling.

### 3.3 Kinetic modeling $\mathrm{RO}D_{\mathrm{Dose}}$ as a function of dose rate for H_2_O and BSA medium

**FIGURE 3:**
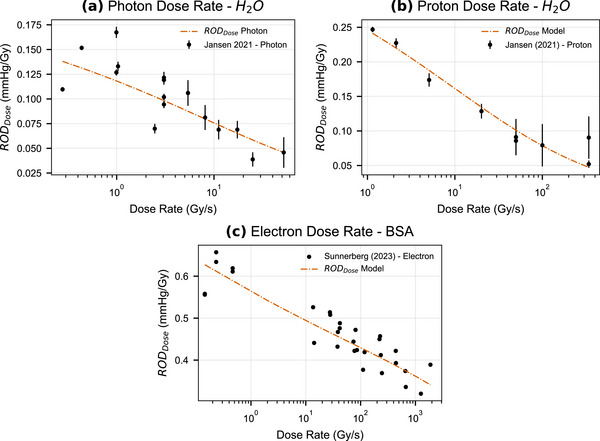
Kinetic model $\mathrm{RO}D_{\mathrm{Dose}}$ prediction for water (a), (b) and BSA (c) medium for variable dose rate. Water was irradiated with photons and protons, whereas BSA was irradiated with electrons. BSA, bovine serum albumin; ROD, radiochemical oxygen depletion.

Published dose rate studies are those from Jansen et al.^6^ and Sunnerberg et al.,^11^ respectively, for water and BSA medium. The kinetic model estimate of $\mathrm{RO}D_{\mathrm{Dose}}$ is fitted to the published dose rate studies (Figure 3), where the $\mathrm{RO}D_{\mathrm{Dose}}$ fits and the experimental data are represented in the Log‐Linear scale that was previously used by Sunnerberg et al.^11^ In addition, Sunnerberg electron studies were performed at the following source surface distances (SSDs) of 60, 100, and 140cm, and with linac repetition rates of 60, 120, 180, and 360Hz. These values correspond to dose rates that vary from 0.14 to 0.46Gy/s for conventional and to 14 to 1500Gy/s for UHDR delivery.

For the Jansen et al.^6^ datasets (photon and proton dose‐rate studies in water), goodness‐of‐fit testing indicated good agreement with the published measurements. Similarly, for the Sunnerberg et al.^11^ dataset (electron irradiation in BSA medium), the reduced $\chi^{2}$ supported good agreement within the stated uncertainties. This shows that the $\mathrm{RO}D_{\mathrm{dose}}$ prediction yields very good agreement with both water and BSA measurements of oxygen consumption for variable dose rates used for radiation beams. In addition, in the Sunnerberg et al.^11^ study, the authors fitted the experimental data with a linear function, using both the mean dose rate and instantaneous dose rate. The $\mathrm{RO}D_{\mathrm{Dose}}$ model prediction given in Figure 3 is consistent with the linear mean dose rate fit.

### 3.4 Kinetic modeling $\mathrm{RO}D_{\mathrm{Dose}}$ as a function of dose rate for in‐vivo mouse studies

In vivo measurements of oxygen consumption by Grilj et al.^12^ are influenced by oxygen diffusion within the tissue during radiation delivery. Consequently, the measured signal reflects not only the intrinsic oxygen consumption (in the absence of diffusion) but also the effects of oxygen diffusion throughout the tissue.

**FIGURE 4:**
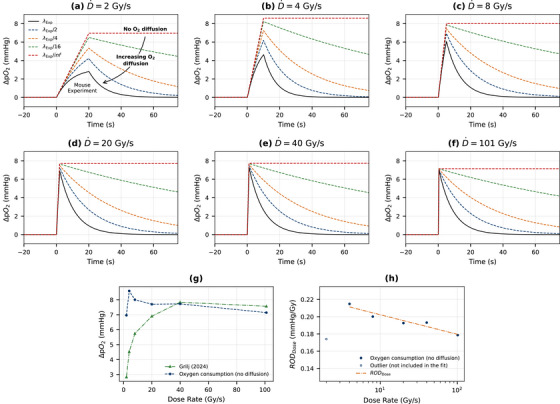
Diffusion corrected $\Delta pO_{2}$ signals from Grilj (2024) paper (a–f), diffusion corrected oxygen consumption (g), and $\mathrm{RO}D_{\mathrm{Dose}}$ fit (h) to new oxygen consumption data ($R^{2}=0.91$).

Figure 4 presents the diffusion‐corrected oxygen consumption results ($\Delta pO_{2}$) for tissue skin at dose rates of 2, 4, 8, 20, 40, and 101Gy/s, as reported in the Grilj (2024)^12^ manuscript (Figure 4a). We show that oxygen consumption values differ significantly when comparing the raw measurements with the diffusion‐corrected values (see Figure 4). Consequently, the convolution approach described in Equation (12) provides a robust method for correcting in vivo oxygen consumption measurements in mice for the effects of oxygen diffusion. In addition, the $\mathrm{RO}D_{\mathrm{Dose}}$ fitting procedure was applied to the newly obtained oxygen consumption data without diffusion. The results indicate an approximately linear relationship when the dose rate is plotted on a logarithmic scale, suggesting that the kinetic ROD model exhibits a predictable dependence on dose rate under these conditions, similar to results presented in Figure 3.

### 3.5 How initial oxygen levels correlate with dose rate during UHDR/FLASH radiation

The initial oxygen dependency of the ROD is described by the Michaelis–Menten function $f(O_{2})$ from Equation (11), where the Michaelis constant is defined as $K_{m}=K\sqrt{\dot{D}}$. The Michaelis constant ($K_{m}$) represents the substrate (oxygen) concentration at which a reaction rate reaches half of its maximum velocity ($V_{\max}$). In this work, the direct correlation between oxygen and dose‐rate is derived at the point corresponding to half the maximum velocity ($V_{\max}/2$), that is when $f\;(O_{2})=0.5$. This condition yields $O_{2}=K\sqrt{\dot{D}}$. Consequently, the functional relationship between dose‐rate and initial oxygen pressure is obtained by inverting the above result, leading to $\dot{D}=\mathrm{Constant}\times {O_{2}}^{2}$, where $\mathrm{Constant}=1/K^{2}$. This midpoint represents the affinity of oxygen for reacting with the radical $e_{\mathrm{aq}}^{•-},$ which directly competes with the reactions involving OH• and $e_{\mathrm{aq}}^{•-}$ (see Equation 5). We can now evaluate the dose rate needed to achieve the same chemical affinity between the two pairs that are in direct competition with each other: ($O_{2}$, $e_{\mathrm{aq}}^{•-}$) and (OH•, $e_{\mathrm{aq}}^{•-}$).

For two different oxygen levels of $O_{2}\left(1\right)$ and $O_{2}\left(2\right)$, we have the following equation:

(13) $$\dot{D}\left(2\right)=\dot{D}\left(1\right)\times {\left(\frac{O_{2}\left(2\right)}{O_{2}\left(1\right)}\right)}^{2}$$

Small changes in the initial oxygen conditions require a significant change in the dose rate to achieve the same chemical affinity. For example, a 20% increase in the initial oxygen concentration, corresponding to $O_{2}\left(2\right)/O_{2}\left(1\right)=1.2$, requires an increase of 44% in the dose rate, corresponding to 1.44 dose rate ratio in Equation (13). Therefore, initial oxygen conditions play a major role in the FLASH effect. As a consequence, it is vital to acquire oxygen tension measurements prior to irradiation to provide an estimate of the chemical affinity between $O_{2}$ and OH•.

### 3.6 How LET affects oxygen consumption and its dependence on initial oxygen pressure

**FIGURE 5:**
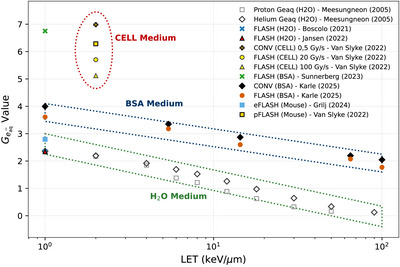
Linear energy transfer (LET) dependency of solvated electron, $G_{e_{\mathrm{aq}}^{•-}}$_._

Using oxygen consumption data from Karle (2025)^22^ across varying initial oxygen pressures, the impact of LET on ROD was assessed for BSA medium. The Michaelis–Menten parameter $V_{\max}$ was obtained from the manuscript (denoted as $g_{\max}$), which allowed for the calculation of the solvated electron *G*‐value, $G_{e_{\mathrm{aq}}^{•-}}$ by dividing by the constant $C_{R}$. Figure 5 shows the $G_{e_{\mathrm{aq}}^{•-}}$ data for different LET. In addition, all $G_{e_{\mathrm{aq}}^{•-}}$ values obtained from the ROD model and presented in Table 3 were also plotted for comparison. Figure 5 also includes, for comparison, previously published $G_{e_{\mathrm{aq}}^{•-}}$ obtained in water for proton and helium beams (Meesungnoen and Jay‐Gerin^23^), and mouse data from Grilj et al.^12^ and Van Slyke et al.^10^

The $G_{e_{\mathrm{aq}}^{•-}}$ values in Figure 5 for the BSA medium appear systematically higher than those for the $H_{2}O$ medium from Boscolo (2021)^21^, Jansen (2022)^8^, and Meesungneon (2005), which agree among themselves. In addition, the respective $G_{e_{\mathrm{aq}}^{•-}}$ values also decrease with increasing LET, for both FLASH and conventional radiation. The $G_{e_{\mathrm{aq}}^{•-}}$ values for the CELL medium also appear systematically higher than those for the BSA and $H_{2}O$ media. The higher $G_{e_{\mathrm{aq}}^{•-}}$ values for BSA and CELL relative to water can be explained by the fact that both the BSA and the CELL medium contain scavengers of the radiolysis reactive species: OH•, $H_{2}O_{2}$, ${\mathrm{HO}}_{2}^{•}$, unlike water. Therefore, the removal of these oxidizing elements by the scavengers will result in a higher concentration of ${\left[e_{\mathrm{aq}}^{•-}\right]}_{\mathrm{SS}}$ in Equation (5), with a subsequent higher $\mathrm{RO}D_{\mathrm{Dose}}$ value for Equation (11), and ultimately a higher $V_{\max}$ and $G_{e_{\mathrm{aq}}^{•-}}$. Consequently, the higher $G_{e_{\mathrm{aq}}^{•-}}$ values observed in Figure 5 for BSA and CELL are a direct consequence of the scavengers present in these medium and not present in water. The mouse $G_{e_{\mathrm{aq}}^{•-}}$ values are very different when comparing the mouse $G_{e_{\mathrm{aq}}^{•-}}$ data from Grilj et al.^12^ and Van Slyke et al.^10^ Further animal studies are needed to identify the origin of this discrepancy. The results presented in Figure 5 indicate that the *G*‐values for solvated electrons predicted by the kinetic ROD model are consistent with published experimental data obtained different particle LETs and different mediums.

## 4 DISCUSSION

The focus of the current work was to develop a kinetic model that could provide an explanation of how ROD depends on:

- Radiation dose rate.
- Initial oxygen pressure, O_2_.

A variety of in vitro (with water, BSA, or CELL medium) and in vivo mouse studies on ROD^6^, ^7^, ^8^, ^9^, ^10^, ^11^, ^12^ were used to evaluate the prediction by the proposed kinetic model of ROD. Equation (10) was used for $\mathrm{RO}D_{\mathrm{Time}}$ and Equation (11) was used for $\mathrm{RO}D_{\mathrm{Dose}}$, which represent separately the ROD dependence in “time” and “dose”, respectively.

The kinetic model estimate for ROD is based on an assessment of how the radiolysis products affect oxygen consumption. The kinetic model assumes that all radiolysis products quickly reach steady state because their main production pathway is the primary radiolysis process, which is radiation dose rate dependent. Under these conditions, steady‐state kinetic analysis can be applied to a smaller sub‐set of reactions consisting of the main production and removal processes. As a consequence, we showed that ROD was dependent on not only the concentration of oxygen but also on the steady state concentration of all the radiolysis products OH•, $e_{\mathrm{aq}}^{•-}$, $O_{2}^{•-}$, ${\mathrm{HO}}_{2}^{•-}$ and their reactions among each other (Equation 1). Since the concentration of oxygen is usually much higher than that of the primary radiolysis species, Equation (1) is approximated by Equation (2), where ROD depends only on the reaction of oxygen with $e_{\mathrm{aq}}^{•-}$. As a consequence, ROD depends directly on the amount of solvated electron available to react with oxygen.

In Jansen et al.,^6^ they hypothesized that the ROD was dependent on the reaction of the solvated electrons with oxygen and the reactive products produced by radiolysis. The results of Jansen et al. for the ROD were shown to be in agreement with the simulation study of Labarbe 2020.^24^ In the follow‐up study by Zhang et al.,^19^ they demonstrated experimentally in water that the dose rate dependence of $H_{2}O_{2}$ and oxygen consumption (not published) is directly related to the “amount” of solvated electrons available during irradiation. They used $N_{2}O$ and $\mathrm{NaN}O_{3}$ to remove solvated electron and demonstrated that the dose rate dependency was then removed. In the current kinetic model, we show that the total concentration of ${\left[e_{\mathrm{aq}}^{•-}\right]}_{\mathrm{SS}}$ available to react with oxygen depends, not only on the oxygen concentration and the dose rate, but also on the concentrations of radiolysis products OH•, ${\mathrm{HO}}_{2}^{•-}$, $H_{2}O_{2}$, which are also affected by other radiolysis products such as $O_{2}^{•-}$.

Other oxygen depletion hypotheses have been proposed to establish an OER model^25^, ^26^, ^27^, ^28^ that is dose rate‐dependent and could be used for in vitro and in vivo experiments. In Petersson (2020),^28^ $\mathrm{RO}D_{\mathrm{Time}}$ was assumed to be proportional to both oxygen and dose rate. The current kinetic model prediction for $\mathrm{RO}D_{\mathrm{Time}}$ provides a generalization of the Petersson (2020)^28^ $\mathrm{RO}D_{\mathrm{Time}}$. For very low oxygen levels, $O_{2}\ll K_{M}=K\sqrt{\dot{D}}$, Equation (10) gives the same approximate functional form as Petersson (2020),^25^ which is $-\frac{\partial [O_{2}]}{\partial t}≅g\dot{D}\left[O_{2}\right]$. From Table 3, we observe that $K_{m}$ varies between 3.4 and 11.4mmHg for in vitro studies, indicating that the Petersson (2020)^28^ approach is only valid for oxygen levels well below 10mmHg, $O_{2}\ll 10\;\mathrm{mmHg}$, which corresponds to hypoxic conditions. In addition, the $\mathrm{RO}D_{\mathrm{Time}}$ estimate in the current work predicts a saturation effect in $\mathrm{RO}D_{\mathrm{Time}}$ for $O_{2}\gg K_{M}$ that is not predicted by the Petersson (2020)^28^ approximation.

Finally, the kinetic model strongly suggests that the radiolysis products, OH•, $e_{\mathrm{aq}}^{•-}$, $O_{2}^{•-}$, ${\mathrm{HO}}_{2}^{•-}$, play an important role in ROD, which is strongly dependent on both the radiation dose rate and the initial oxygen pressure. In addition, the model also predicts that if the initial oxygen pressure is too high ($O_{2}\gg K_{M}=K\sqrt{\dot{D}}$), the dose rate dependence is lost, and the FLASH effect is unlikely to be easily achieved. Therefore, a key factor in correlating the magnitude of the FLASH effect within the same organ but for different mice is the Michaelis constant, $K_{M}=K\sqrt{\dot{D}}$, which determines the chemical affinity between the initial oxygen pressure and the amount of radiolysis products produced at a certain dose rate. Different mice may have slightly different levels of oxygen in the same organ, which can lead to the variability in the observed FLASH effect, if the chemical affinity is not taken into account.

The primary limitation of this study is that the kinetic ROD model was constructed using water radiolysis as its foundation. Upon radiolysis of pure water, oxygen is primarily consumed through reduction by solvated electrons $e_{\mathrm{aq}}^{•-}$ and hydrogen atoms H•, producing superoxide $O_{2}^{•-}$ and its conjugate acid, the hydroperoxyl radical ${\mathrm{HO}}_{2}^{•}$. In the presence of organic molecules (RH), the reaction pathways are altered. The hydroxyl radical OH• can oxidize RH, generating organic radicals, R• that react with oxygen to form peroxyl radicals, ROO•. Therefore, the oxygen consumption equation (Equation 2) must be modified to account for the effects of organic radicals, as described by El Khatib (2022)^29^:

(14) $$\frac{\partial \left[O_{2}\right]}{\partial t}≅-k_{R5}.\left[O_{2}\right].\left[e_{\mathrm{aq}}^{•-}\right]-k_{R}.\left[O_{2}\right].\left[R•\right]$$

In Equation (14), oxygen consumption is driven by two radicals: the solvated electron and the organic radical. The key distinction is that R• is not generated directly by radiation but rather through the reaction of RH with OH•. Consequently, in biological environments, oxygen consumption occurs through two primary pathways: (1) direct reduction of oxygen by solvated electrons, yielding superoxide, and (2) indirectly via the oxidation of organic molecules by hydroxyl radicals to form organic radicals (R•), which then react further with oxygen. At conventional radiotherapy dose rates, these reactions occur independently. In contrast, under UHDR/FLASH conditions, the high local densities of $e_{\mathrm{aq}}^{•-}$, R•, and their secondary species $O_{2}^{•-},$ ROO• promote cross‐reactions between them, effectively diminishing the overall radiation‐induced toxicity, which is dose rate dependent. In Equation (14), both $e_{\mathrm{aq}}^{•-}$ and R• must exhibit a Michaelis–Menten behavior in order to fit experimental data.^6^, ^7^, ^8^, ^9^, ^10^, ^11^, ^12^ Therefore, an expected general solution to this equation remains the Michaelis–Menten function provided by Equations (10) and (11).

## 5 CONCLUSION

A systematic review of all published ROD studies was performed and used as the basis for testing the novel kinetic model for ROD. We have developed a novel kinetic model for ROD and tested it against published experimental and simulation data. The radiolysis products, OH•, $e_{\mathrm{aq}}^{•-}$, $O_{2}^{•-}$, ${\mathrm{HO}}_{2}^{•-}$, were shown to play an important role in ROD leading to (1) the dose rate dependence and (2) the initial oxygen dependence in ROD.

## CONFLICT OF INTEREST STATEMENT

The authors declare no conflicts of interest.

## References

[1] Favaudon V , Caplier L , Monceau V , et al. Ultrahigh dose rate FLASH irradiation increases the differential response between normal and tumor tissue in mice. Sci Transl Med. 2014;6(245):245ra93. doi:10.1126/scitranslmed.3008973 25031268

[2] Seco J , Deasy JO , Das IJ . FLASH radiotherapy: Paradigm shift or a passing fad?. Med Phys. (2025);52(6):3504‐3508. doi:10.1002/mp.17807 40184041

[3] Town CD . Effect of high dose rates on survival of mammalian cells. Nature. 1967;215:847‐848. doi:10.1038/215847a0 6049731

[4] Berry R , Hall EJ , Forster DW , Storr TH , Goodman MJ . Survival of mammalian cells exposed to x rays at ultra‐high dose‐rates. Br J Radiol. 1969;42(494):102‐107. doi:10.1259/0007-1285-42-494-102 4975207

[5] Weiss H , Epp ER , Heslin J , Ling CC , Santomasso A . Oxygen depletion in cells irradiated at ultra‐high dose‐rates and at conventional dose‐rates. Int J Radiat Biol Relat Stud Phys Chem Med. 1974;26(1):17‐29. 4607987 10.1080/09553007414550901

[6] Jansen J , Knoll J , Beyreuther E , et al. Does FLASH deplete oxygen? Experimental evaluation for photons, protons, and carbon ions. Med Phys. 2021;48(7):3982‐3990. doi:10.1002/mp.14917 33948958

[7] Cao X , Zhang R , Esipova TV . Quantification of oxygen depletion during FLASH irradiation in vitro and in vivo. Int J Radiat Oncol Biol Phys. 2021;111(1):240‐248. doi:10.1016/j.ijrobp.2021.03.056 33845146 PMC8338745

[8] Jansen J , Beyreuther E , García‐Calderón D , et al. Changes in radical levels as a cause for the FLASH effect: impact of beam structure parameters at ultra‐high dose rates on oxygen depletion in water. Radiother Oncol. 2022;175:193‐196. doi:10.1016/j.radonc.2022.08.024 36030933

[9] Boscolo D , Krämer M , Fuss MC , Durante M , Scifoni E . Impact of target oxygenation on the chemical track evolution of ion and electron radiation. Int J Mol Sci. 2020;21(2):424. doi:10.3390/ijms21020424 31936545 PMC7014692

[10] Van Slyke AL , El Khatib M , Velalopoulou A , et al. Oxygen monitoring in model solutions and in vivo in mice during proton irradiation at conventional and FLASH dose rates. Radiat Res. 2022;198(2):181‐189. doi:10.1667/RADE-21-00232.1 35640166 PMC10176203

[11] Sunnerberg JP , Zhang R , Gladstone DJ , Swartz HM , Gui J , Pogue BW . Mean dose rate in ultra‐high dose rate electron irradiation is a significant predictor for O2consumption and H2O2yield. Phys Med Biol. 2023;68(16):165014. doi:10.1088/1361-6560/ace877 PMC10405361 37463588

[12] Grilj V , Leavitt RJ , El Khatib M , et al. In vivo measurements of change in tissue oxygen level during irradiation reveal novel dose rate dependence. Radiother Oncol. 2024;201:110539. doi:10.1016/j.radonc.2024.110539 39299575

[13] Wren JC , Ball JM . LIRIC 3.2 an updated model for iodine behaviour in the presence of organic impurities. Radiat Phys Chem. 2001;60:577. doi:10.1016/S0969-806X(00)00385-6

[14] Buxton GV , Greenstock CL , Helman WP , Ross AB . Critical review of rate constants for reactions of hydrated electron, hydrogen atoms and hydroxyl radicals in aqueous solution. J Phys Chem Ref Data. 1988;17:513 . doi:10.1063/1.555805

[15] Elliot AJ , Buxton GV . Temperature dependence of the reactions OH+O^−^ _2_ and OH+HO_2_ in water up to 200 1C. J Chem Soc Faraday Trans. 1992;88(17):2465. doi:10.1039/FT9928802465

[16] Elliot AJ . Rate constants and G‐values for the simulation of the radiolysis of light water over the range 0–300 °C. AECL‐11073, COG‐94‐167. Atomic Energy of Canada Limited, Chalk River Nuclear Labs, 1994.

[17] Sunaryo GR , Katsumura Y , Ishigure K . Radiolysis of water at elevated temperatures‐III. Simulation of radiolytic products at 25 and 250 1C under the irradiation with gamma rays and fast neutrons. Radiat Phys Chem. 1995;45:703. doi:10.1016/0969-806X(94)00084-W

[18] Joseph JM , Choi BS , Yakabuskie P , Wren JC . A combined experimental and model analysis on the effect of pH and O2(aq) on γ‐radiolytically produced H2 and H2O2. Radiat Phys Chem. 2008;77(9):1009‐1020. doi:10.1016/j.radphyschem.2008.06.001

[19] Zhang T , Stengl C , Derksen L , et al. Analysis of hydrogen peroxide production in pure water: ultrahigh versus conventional dose‐rate irradiation and mechanistic insights. Med Phys. 2024;51(10):7439‐7452. doi:10.1002/mp.17335 39092902

[20] Ayene IS , Koch CJ , Krisch RE . Simulation of the cellular oxygen effect with an SV40 DNA model system using DNA strand breaks as an endpoint. Radiat Res. 1996;146:501‐509. doi:10.2307/3579550 8896576

[21] Boscolo D , Scifoni E , Durante M , Krämer M , Fuss MC . May oxygen depletion explain the FLASH effect? A chemical track structure analysis. Radiother Oncol. 2021;162:68‐75. doi:10.1016/j.radonc.2021.06.031 34214612

[22] Karle C , Liew H , Tessonnier T , et al. Oxygen consumption measurements at ultra‐high dose rate over a wide LET range. Med Phys. 2025;52(2):1323‐1334. doi: 10.1002/mp.17496 39504410 PMC11788059

[23] Meesungnoen J , Jay‐Gerin JP . High‐LET radiolysis of liquid water with 1H+, 4He2+, 12C6+, and 20Ne9+ ions: effects of multiple ionization. J Phys Chem A. 2005;109(29):6406‐6419. doi:10.1021/jp058037z 16833985

[24] Labarbe R , Hotoiu L , Barbier J , Favaudon V . A physicochemical model of reaction kinetics supports peroxyl radical recombination as the main determinant of the FLASH effect. Radiother Oncol. 2020;153:303‐310. doi:10.1016/j.radonc.2020.06.001 32534957

[25] Pratx G , Kapp DS . A computational model of radiolytic oxygen depletion during FLASH irradiation and its effect on the oxygen enhancement ratio. Phys Med Biol. 2019;64(18):185005. doi:10.1088/1361-6560/ab3769 31365907

[26] Taylor E , Hill RP , Létourneau D . Modeling the impact of spatial oxygen heterogeneity on radiolytic oxygen depletion during FLASH radiotherapy. Phys Med Biol. 2022;67(11). doi:10.1088/1361-6560/ac702c 35576920

[27] Hu A , Qiu R , Wu Z , Zhang H , Li WB , Li J . A computational model for oxygen depletion hypothesis in FLASH effect. Radiat Res. 2022;197(2):175‐183. doi:10.1667/RADE-20-00260.1 34739052

[28] Petersson K , Adrian G , Butterworth K , McMahon SJ . A quantitative analysis of the role of oxygen tension in FLASH radiation therapy. Int J Radiat Oncol Biol Phys. 2020;107(3):539‐547. doi:10.1016/j.ijrobp.2020.02.634 32145319

[29] El Khatib M , Van Slyke AL , Velalopoulou A , et al. Ultrafast tracking of oxygen dynamics during proton FLASH. Int J Radiat Oncol Biol Phys. 2022;113(3):624‐634. doi:10.1016/j.ijrobp.2022.03.016 35314293 PMC9250619

